# Recurrent Cochlear Implant Electrode Exposure in a Smoker With Prior Canal Wall Mastoidectomy: A Case Report

**DOI:** 10.7759/cureus.108505

**Published:** 2026-05-08

**Authors:** Loga S Iyer, Anita Jeyakumar

**Affiliations:** 1 Otolaryngology, Northeast Ohio Medical University, Rootstown, USA; 2 Otolaryngology-Head and Neck Surgery, HEARS Otology, Akron, USA

**Keywords:** canal wall down mastoidectomy, cochlear implantation, electrode exposure, implant failure, smoking, soft-tissue complications, wound healing

## Abstract

Cochlear implantation (CI) is an established and effective surgical intervention for patients with severe to profound sensorineural hearing loss. The procedure involves placement of an internal receiver-stimulator in a postauricular subperiosteal pocket with an electrode advanced through the mastoid and facial recess into the cochlea. Long-term device function depends on secure fixation and durable soft tissue coverage over both the implant and extracochlear electrode segment to prevent skin breakdown, infection, and electrode exposure. Although electrode exposure is uncommon, it represents a serious complication that can compromise implant integrity and frequently necessitates revision surgery or explantation.

Patients with prior history of canal wall down (CWD) mastoidectomy are at increased risk of this complication given the altered mastoid anatomy, reduced soft tissue bulk, and compromised vascular supply. In addition, a history of chronic tobacco use further exacerbates these risks by inducing vasoconstriction, reducing tissue oxygenation, and impairing collagen synthesis increasing the likelihood of postoperative skin breakdown. We present a case of recurrent CI electrode exposure in an elderly patient with a remote history of CWD mastoidectomy and chronic tobacco use, highlighting the compounded impact of these factors on postoperative healing and device viability.

## Introduction

Cochlear implantation (CI) is a widely accepted and common procedure for severe to profound sensorineural hearing loss, with low overall complication rates. However, postoperative wound complications, including flap necrosis, device or electrode exposure, and wound dehiscence, remain clinically significant due to risk of infection, loss of device function, and need for revision surgery or explantation [1]. Maintenance of a robust soft tissue envelope over the receiver-stimulator and extracochlear electrode is critical for long-term implant viability [2].

When electrode exposure does occur, it presents a difficult clinical challenge. The exposed device is at risk for bacterial colonization and biofilm formation, which can progress to deep infection and ultimately require device explantation. Management options range from local wound care and antibiotic therapy for minor cases to surgical revision with soft-tissue flap coverage and, in refractory or infected cases, full device explantation. Successful salvage depends heavily on the adequacy of local tissue perfusion and the absence of ongoing factors that impair wound healing [2]. 

Prior canal wall down (CWD) mastoidectomy is performed for advanced or recurrent cholesteatoma and failed canal wall up surgery and involves the removal of the superior and posterior bony external auditory canal walls, creating a single open cavity lined by keratinizing epithelium rather than vascularized mucosa [3]. This altered anatomy leaves the CI electrode overlain by a thin epithelial layer over cortical bone with no intervening vascularized tissue and disrupts the occipital and posterior auricular arterial supply from prior canalplasty. These structural factors may impede durable wound closure over an implanted device. Hunter et al. demonstrated in a systematic review of 424 patients that CI performed in a maintained CWD cavity carried an approximately 30% complication rate, substantially higher than rates achieved with cavity obliteration or subtotal petrosectomy [4].

Chronic tobacco use is a well-established risk factor for impaired wound healing across surgical disciplines. Nicotine-mediated vasoconstriction, carbon monoxide-induced tissue hypoxia, and suppressed fibroblast and immune cell function collectively compromise wound repair [5]. In a meta-analysis of over 479,000 patients, active smokers had significantly elevated odds of tissue necrosis (OR: 3.60; 95% CI: 2.62-4.93) and combined wound complications (OR: 2.27; 95% CI: 1.82-2.84) compared with nonsmokers [5]. In head and neck reconstructive surgery, tobacco use was independently associated with a 74% increased odds of wound disruption [6].

Advanced age further compounds these risks through progressive microvascular decline and reduced immune surveillance [7]. Comorbidities prevalent in elderly patients, including diabetes mellitus, malnutrition, peripheral vascular disease, and chronic corticosteroid use, each independently impair soft tissue healing and should be assessed as part of preoperative risk stratification in CI candidates [8]. Despite recognition of these individual risk factors, limited literature addresses their combined impact on cochlear implant outcomes. This case is reported to highlight the challenges of achieving durable soft tissue coverage in cochlear implant recipients with extensive prior otologic surgery and impaired wound healing and to underscore considerations for surgical planning and patient counseling in this high-risk population.

## Case presentation

A 75-year-old male patient with a complex left otologic history presented with asymmetric hearing loss and significant tinnitus. He reported normal hearing during childhood but developed an "ear tumor" as a young adult, most consistent with cholesteatoma. Approximately 60 years prior, he underwent left ear surgery resulting in complete left-sided hearing loss, followed by a second left ear surgery. His surgical history was consistent with a prior CWD mastoidectomy, and examination revealed a left mastoid bowl. He denied vertigo or balance symptoms. The patient reported a chronic history of tobacco use. No major comorbidities, including diabetes mellitus, peripheral vascular disease, or immunosuppressive therapy, were documented in the available preoperative record, though formal nutritional assessment was not performed given the retrospective nature of this report.

The patient had not previously used amplification and experienced no perceived benefit from trials of a CROS hearing aid or a softband bone-anchored hearing device. Given his hearing profile, he underwent left CI after preoperative counseling regarding elevated wound complication risk related to his prior CWD mastoidectomy and ongoing tobacco use. Preoperative imaging demonstrated a normal cochlea and internal auditory canals.

**Figure 1:**
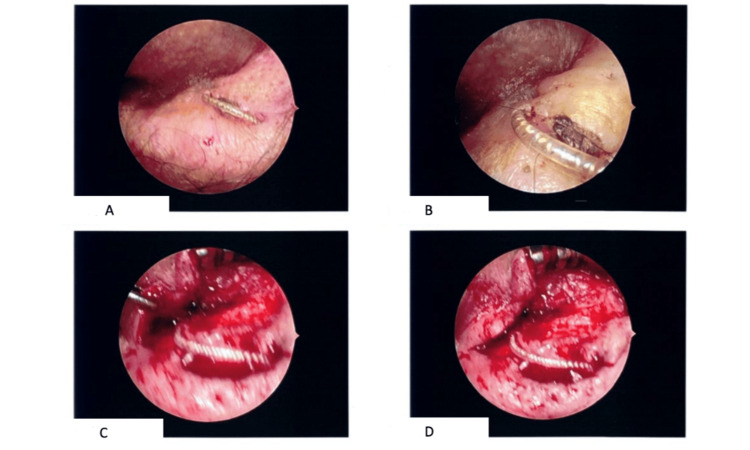
Intraoperative reconstruction of electrode exposure using a temporal muscle flap (A & B) Clinical images demonstrating electrode exposure at the skin surface overlying thinned soft tissue. Key findings visible include the extruded electrode lead at the wound surface and surrounding skin atrophy consistent with chronic ischemic change. (C & D) Intraoperative images showing the rotational temporal muscle flap used to reinforce soft tissue coverage over the electrode, providing vascularized tissue bulk as a protective barrier. The superior margin of the flap is rotated over the exposed electrode; the stippled margin demarcates the flap-recipient tissue interface

**Figure 2:**
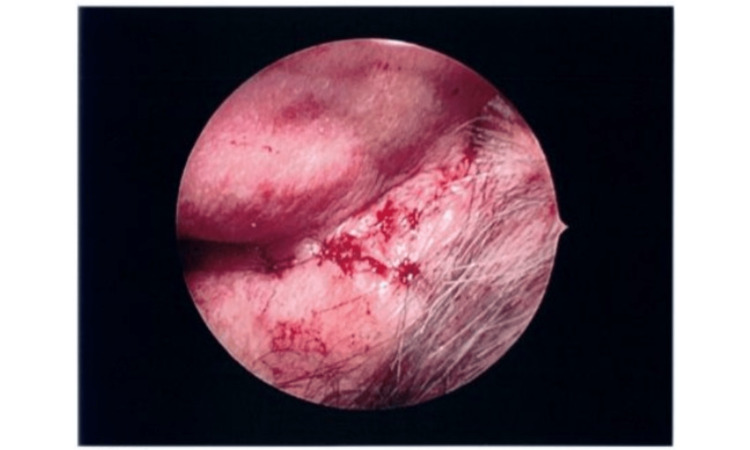
Postoperative appearance after revision surgery with temporal muscle flap reconstruction The electrode is covered beneath intact skin closure with satisfactory short-term healing and no evidence of recurrent exposure at the time of this photograph. Note the well-approximated incision line and absence of erythema or fluctuance at this early postoperative timepoint

Approximately one year postoperatively, the patient developed exposure of the CI electrode, necessitating revision surgery (Figure 1). Intraoperatively, thinning of the overlying soft tissue was noted. The area was reinforced with a rotational temporal muscle flap to serve as a protective barrier (Figures 1C-1D). Short-term postoperative healing appeared satisfactory (Figure 2). Despite revision, the patient developed recurrent skin thinning with re-exposure of the electrode, associated with progressive pain and subsequently early signs of superinfection. Plastic surgery consultation raised concerns regarding soft tissue integrity and long-term device viability. Given repeated exposure and failure of primary salvage, the patient underwent explantation of the left cochlear implant. The wound healed without further complication, and the patient returned to pre-implant functional status with complete left-sided hearing loss.

## Discussion

CI is generally safe, but wound complications such as flap necrosis, dehiscence, and electrode exposure remain recognized risks, particularly in complex surgical environments [1]. Prior CWD mastoidectomy creates a potentially vulnerable soft tissue bed with absent vascularized mucosa, disrupted regional arterial supply, and an open epithelialized cavity that may impede durable wound closure over an implanted device [3,4]. Hunter et al. demonstrated that CI in CWD cavities is associated with an approximately 30% complication rate when the open cavity is maintained, and that electrode extrusion has been reported in a substantial proportion of such cases [4]. In a series examining electrode array extrusion specifically, the majority of adult patients with this complication have been found to have a prior history of open-cavity mastoidectomy, underscoring the primacy of this anatomic risk factor [9].

Electrode exposure in the cochlear implant literature is reported at rates ranging from 1% to over 5% across large series and may present acutely or in a delayed fashion, often months to years after implantation [2,9]. In published series, revision surgery without explantation succeeds in only approximately 40% of major skin flap complications, with eventual explantation required in the majority of refractory cases [10]. The present case followed this pattern, with the first electrode exposure occurring at approximately 12 months postoperatively. Initial flap salvage achieved short-term wound closure but was insufficient to sustain durable coverage in a chronically compromised soft tissue environment.

Tobacco use independently worsens wound healing through various pathophysiologic mechanisms. Smoking introduces nicotine, carbon monoxide, and other toxic compounds that cause vasoconstriction, reduce tissue oxygenation, and impair fibroblast and inflammatory cell function [5]. These effects are observed across multiple surgical disciplines, where smokers face significantly higher rates of wound dehiscence, delayed healing, and surgical site infection. In head and neck reconstructive surgery, active tobacco use was independently associated with significantly elevated wound disruption and reoperation risk [6]. A retrospective cohort study of patients undergoing percutaneous auditory osseointegrated implant placement found that tobacco users experienced higher rates of soft tissue reactions than nonsmokers (24.5% vs. 6.8% early; 40.9% vs. 19.2% long-term) [11]. In the present case, the combination of altered soft tissue from prior CWD mastoidectomy and the systemic wound-healing impairment from chronic tobacco use may have created an environment less favorable for wound healing, with the residual flap dependent on collateral perfusion in exactly the territory most susceptible to nicotine-induced vasoconstriction.

It must be acknowledged that this is a single case report, and no definitive causal attribution can be made to any individual risk factor. The patient's advanced age introduces additional wound-healing considerations not formally characterized in this retrospective report. Comorbidities prevalent in elderly patients, including diabetes mellitus (OR: 1.53 for surgical site infection), malnutrition, peripheral vascular disease, and chronic corticosteroid use, may have contributed to the outcome and cannot be excluded as cofactors [8]. Smoking should therefore be interpreted as one contributing factor within a likely multifactorial risk profile.

The decision to proceed with explantation rather than a second salvage revision was informed by the recurrent nature of the exposure, early superinfection, and multidisciplinary consensus that further reconstruction was unlikely to achieve durable coverage given the patient's wound environment. Published management frameworks generally favor explantation over repeat revision in cases of recurrent failure, particularly in the setting of active infection and absence of viable local tissue [12,13]. Smoking represents a modifiable risk factor, and preoperative cessation counseling initiated at least four weeks prior to surgery is associated with significant reductions in postoperative wound complications and should be routine in CI candidacy evaluation [5,14]. For patients with prior CWD mastoidectomy, consideration of cavity obliteration or subtotal petrosectomy prior to implantation may substantially reduce complication risk and warrants discussion during surgical planning [4].

## Conclusions

This case highlights the increased risk of wound complications in patients with both altered mastoid anatomy and impaired wound healing capacity. The occurrence of recurrent cochlear implant electrode exposure progressing to explantation in the combined setting of prior CWD mastoidectomy and chronic tobacco use underscores the compounded effect of structural and biologic risk factors on device viability. Advanced age and potentially uncharacterized comorbidities likely also contributed, and the findings should be interpreted as suggestive of a multifactorial risk profile rather than attributable to any single variable. Recognizing these factors preoperatively may guide surgical decision-making, optimize patient counseling, and help mitigate preventable complications in similar complex cases.
